# Effect of Chinese herbal medicine formula on progression-free survival among patients with metastatic colorectal cancer: Study protocol for a multi-center, double-blinded, randomized, placebo-controlled trial

**DOI:** 10.1371/journal.pone.0275058

**Published:** 2022-12-16

**Authors:** Qiaoling Wang, Zhuohong Li, Jing Guo, Wenyuan Li, Fengming You

**Affiliations:** 1 Department of Scientific Research, Hospital of Chengdu University of Traditional Chinese Medicine, Chengdu, Sichuan Province, China; 2 Department of Oncology, Hospital of Chengdu University of Traditional Chinese Medicine, Chengdu, Sichuan Province, China; 3 Evidence-Based Medicine Center, Hospital of Chengdu University of Traditional Chinese Medicine, Chengdu, Sichuan Province, China; Tata Memorial Centre, INDIA

## Abstract

**Introduction:**

Metastatic colorectal cancer (mCRC) causes high cancer-related morbidity and mortality worldwide. Although chemotherapy and targeted agents treatment improve median survival and 5-year survival rates, there is only one-third of patients who adhere to treatment protocol until the pause of disease progression. Hezhong granule is a traditional Chinese herbal formula used for mCRC, which has shown good potential in alleviating the adverse effects of chemotherapy, enhancing the effectiveness of chemotherapy, and improving the quality of life. Therefore, the purpose of the study is to further validate the clinical efficacy and safety of the Chinese herbal medicine formula named Hezhong (HZ) in combination with standard chemotherapy and cetuximab (CET) or bevacizumab (BV) for treating mCRC.

**Methods:**

In this multi-center, randomized, double-blinded, placebo-controlled trial, 360 eligible mCRC patients who will be randomly assigned to Hezhong or placebo group with a 1: 1 ratio. Participants in the Hezhong group will receive standard chemotherapy and CET or BV plus Hezhong Granule until the pause of disease progression, death, the exhibition of intolerable toxicity, or up to 6 months, while the placebo group will treat with standard chemotherapy and CET or BV plus placebo. The primary endpoint is progression-free survival (PFS). The secondary endpoints are overall survival (OS), objective response rate (ORR), safety, quality of life years (QOL), and chemotherapy-induced nausea and vomiting (CINV).

**Expected results:**

The expected results of this trial are to improve the PFS and QOL of patients with mCRC and provide evidence-based recommendations for the treatment of mCRC with traditional Chinese medicine in China.

**Trial registration:**

The trial has been registered with the Chinese Clinical Trial Registry (ChiCTR). The trial registration number was ChiCTR2100041643.

## Introduction

Colorectal cancer (CRC) is one of the most commonly diagnosed cancer worldwide and the second most deadly cancer leading to more than 935 000 deaths annually [1, 2]. The incidence rate of colorectal cancer has been increasing year by year and now it ranks third among malignant tumors. It is the second most common cancer cause of death in China [3]. According to the data on Cancer statistics in China, colorectal cancer is expected to account for 12.28% of all new malignancies in China in 2022 [4]. Approximately 20%-30% of colorectal cancer patients present with advanced stages at the time of diagnosis, and patients in the early stages of the disease also have a 25%-50% chance of developing metastases [5, 6]. In recent years, the use of cytotoxic chemotherapies and targeted agents therapies led to a significant increase in overall survival, but mCRC remains incurable in most cases [4]. Drug resistance and chemotherapy-related toxic side effects are the main causes for the failure or discontinuation of chemotherapy in mCRC. The effectiveness of the first-line chemotherapic agents for primary treatment of mCRC is only 40%-60%. The efficiency of second-line chemotherapic agents is less than 30%, and the efficiency of chemotherapy in patients who are not successful with second-line chemotherapy is even lower, usually less than 15% [7]. For this group of patients who have cancer progression after second-line chemotherapy, effective treatment is lacking [8]. Furthermore, with the increase of chemotherapy cycles, the toxic side effects of chemotherapy also increase, and therefore, most patients discontinue chemotherapy because of intolerance to the toxic side effects [9]. Consequently, prolonging survival, reducing the rate of recurrence and metastasis, and improving the quality of life of patients with mCRC are problematic, and hence, alternative treatments are sought.

Chinese herbal medicine (CHM) has become a complementary alternative therapy in the treatment of cancers including CRC and its use is widely accepted in China [10, 11]. CHM was shown to inhibit colon tumor formation, proliferation, migration, induce apoptosis and modulate angiogenesis of CRC cells [12–14]. In addition, many clinical trials showed that CHM used in combination with chemotherapies reduced toxicity induced by the chemotherapy, enhanced immune function, improved quality of life, and maintained safety [15–18]. Hezhong (HZ) granule is a Chinese herbal formula composed of eight herbs including zingibers, ginseng, Scutellaria baiealensis, Chinese goldthread rhizome, Indian bread, Euodia rutaecarpa, and Pinellia ternata, which has been used in the treatment of mCRC for many years as an empirical formula [19–21]. Some studies have shown that Hezhong and its components can inhibit the release of 5-hydroxytryptamine3 and substance P from chromaffin cells of the gastrointestinal mucosa, reduce the level of tryptophan hydroxylase, down-regulate the expression of the neurokinin-1 receptor, inhibit gastric secretion and pepsin activity, etc.; thereby promoting the repair of gastric mucosa, inhibiting vomiting center and reducing the reactions of gastrointestinal [22–27]. However, many of the previous studies are deficient in study design, sample size, and evidence to determine whether Hezhong granule can benefit patients with mCRC. Therefore, the current study is to observe the efficacy and safety of the Chinese herbal medicine formula, Hezhong (HZ) granule when combined with standard chemotherapy and molecular targeted therapy in patients with mCRC.

### Study design and methods

**Fig 1:**
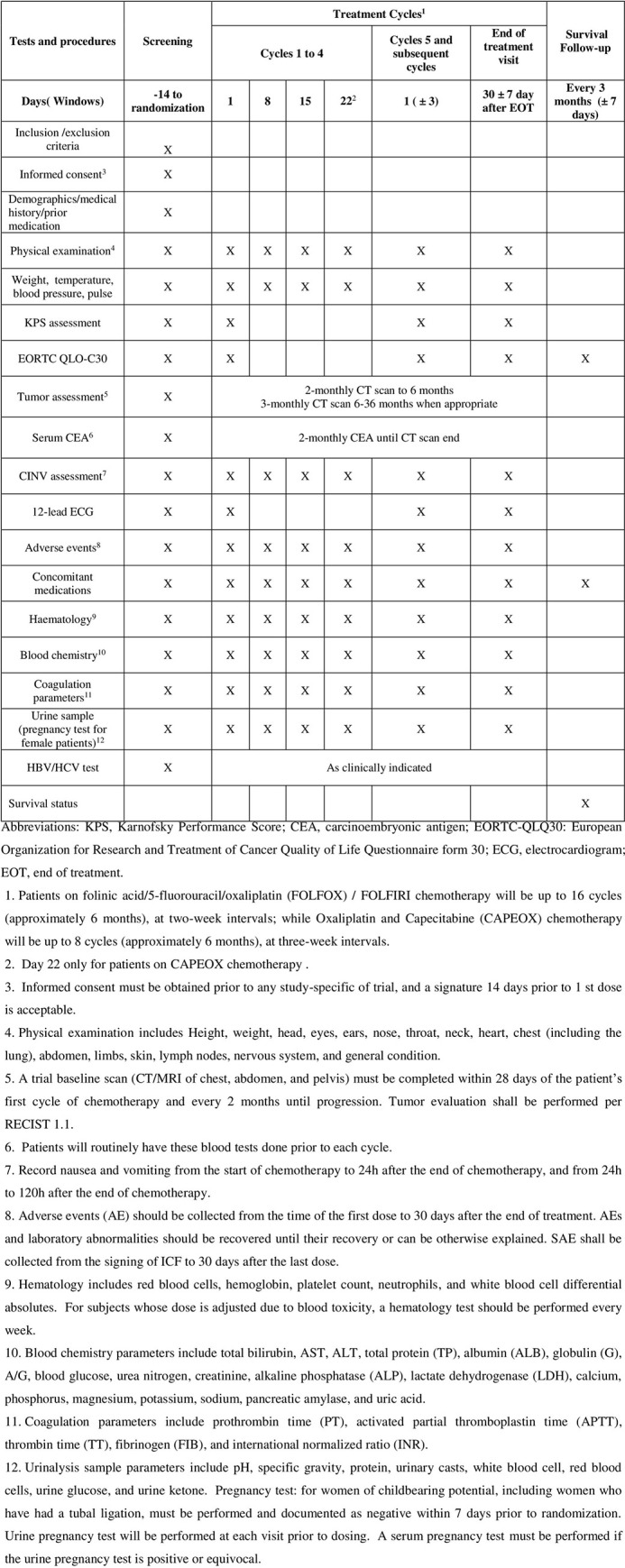
SPIRIT schedule of tests and procedures.

**Fig 2:**
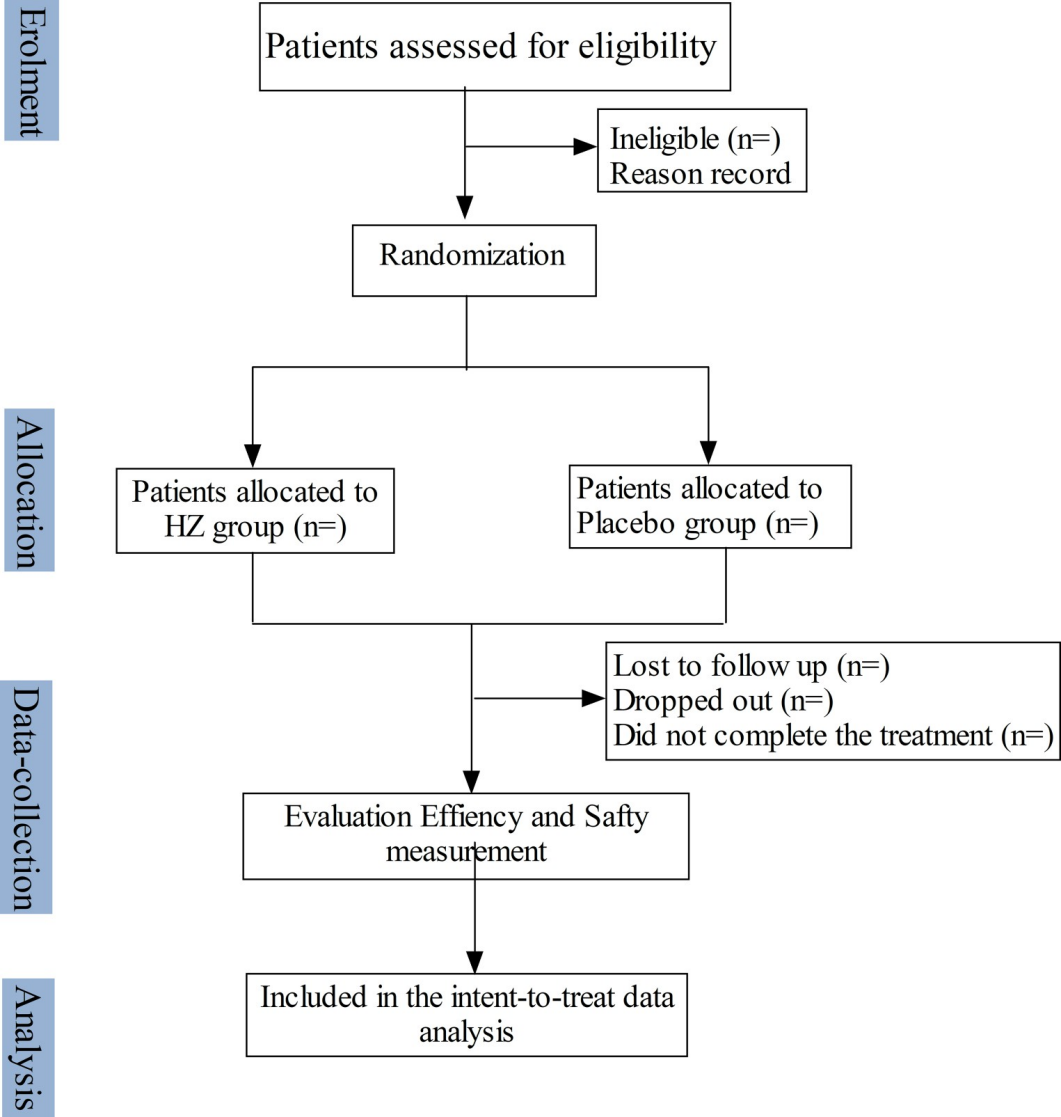
Flow chart.

This study is a multi-center, randomized, double-blinded, placebo-controlled clinical trial and involves twelve clinical sites in China. A total of 360 mCRC patients will be randomly divided into two groups (1:1). The SPIRIT schedule of tests and procedures for this study can be found in Fig 1. The control group will receive a placebo combined with mFOLFOX6/FOLFIRI/CAPEOX and CET or BV a standard first-line treatment for metastatic colorectal cancer. The treatment group will receive HZ granule in combination with mFOLFOX6/FOLFIRI/CAPEOX and CET or BV (Flow chart. Fig 2). The study protocol obeyed the Standard Protocol Items for Randomized Trials statement, the Declaration of Helsinki, and the Good Clinical Practice guidelines. The study has been registered in the Chinese Clinical Trial Registry (ChiCTR) and the registration number was ChiCTR2100041643.

### Study objectives

Our primary objective is to validate the PFS of participants with mCRC who will be randomized to the HZ or placebo group. PFS is defined as the time from randomization to disease progression or death from any cause, whichever occurred earlier. Secondary objectives are to analyze overall survival (OS, time from the start of therapy to death from any cause), objective response rate (ORR, proportion of patients with confirmed complete or partial response), safety, quality of life (QOL) [28], and chemotherapy-induced nausea and vomiting (CINV) [29], comparing various indicators between two groups.

Tumor assessments will be done every 2 months according to the standard of Response Evaluation Criteria in Solid Tumors version 1.1 (RECIST 1.1) [30] for the initial 6 months, and then every 3 months until disease progression or death during the follow-up time. The European Organization for Research on Treatment of Cancer (EORTC) QLQ-C30 scale will be used to assess the quality of life including functions, symptoms, and living conditions through linear conversion to a percentage system at baseline and every chemotherapy cycle, and thereafter until the end of the follow-up period [31]. Adverse events (AEs) will be recorded during the study based on the incidence, severity, and outcomes. Safety assessments are based on Common Terminology Criteria Adverse Events Version 4.0 (CTCAE v4.0) [32].

### Study setting

The study plans to conduct in twelve medical institutions in China. The hospital at the Chengdu University of Traditional Chinese Medicine lead the research, and other participating units are West China Hospital of Sichuan University, Sichuan Provincial Hospital of Integrated Traditional Chinese and Western Medicine, Chengdu Hospital of Integrated Traditional Chinese and Western Medicine, Affiliated Hospital of North Sichuan Medical College, Guangyuan Central Hospital, Jintang County Hospital of Traditional Chinese Medicine, Yanjiang District Hospital of Ziyang City, Cangxi County People’s Hospital, Cangxi County Hospital of Traditional Chinese Medicine, Renshou County People’s Hospital, and Pengzhou Hospital of Traditional Chinese Medicine.

### Eligibility criteria

Participants meet the criteria during the screening examination to be eligible to participate in the study.

### Inclusion criteria

1. Patients who had histologically or cytologically confirmed adenocarcinoma of the colon or rectum that was metastatic or locally advanced with stage IV;
2. Patients who underwent imaging examination (PET-CT, CT, MRI, bone scan, X-ray) that confirmed the presence of measurable lesions defined by RECIST 1.1;
3. Patients with the age ranged of 18 to 75 years;
4. Patients with Karnofsky Performance Score (KPS) of ≥50 [33];
5. Patients who had no major surgery or open biopsy within 4 weeks of random assignment;
6. Patients who agree to receive first-line chemotherapy combined with targeted therapy (Patients may have received previous chemotherapy and/or chemoradiation per institutional standard of care. The last adjuvant therapy must have been concluded more than 6 months ago);
7. Patients with adequate organ and bone marrow functions as defined below: absolute neutrophil count (ANC) of >1.5×10^9^/L; platelets count of >100×10^9^/L; hemoglobin level of ≥90 g/L; total bilirubin level of ≤1.5 times the upper limit of normal (ULN); AST (SGOT) or ALT (SGPT) level of ≤2.5 times the ULN (or ≤5 times the ULN if it was attributable to liver metastases); Urine protein/creatinine ratio (UPCR) of <1.0.
8. Patients with the expected survival period of more than 12 weeks;
9. Patients are able to understand and willing to sign the written informed consent form.

### Exclusion criteria

1. Patients who had histologically confirmed mixed adenosquamous carcinoma with squamous cells as the main component;
2. Patients with other concurrent malignancies or had prior treatment for other carcinomas within the last 5 years, except cured carcinoma in situ of the cervix or non-melanoma skin cancer or superficial bladder tumors;
3. Patients who are pregnant or breastfeeding (a serum or urine pregnancy test is performed on all female patients who are of childbearing potential within 14 days prior to study entry);
4. Patients with prior history of hypertensive crisis, hypertensive encephalopathy, or uncontrolled hypertension (systolic blood pressure of >150mmHg, or diastolic blood pressure of >100mm Hg after anti-hypertensive therapy);
5. Patients who experienced vital organ failure or with other serious diseases including, but not limited to, coronary heart disease, cardiovascular diseases, or myocardial infarction within 12 months before being included in the study; severe neurological or psychiatric history; severe infection; active disseminated intravascular coagulation; serious diseases of the urinary system and digestive system;
6. Patients who had a combination of active hepatitis, pneumonia, and other serious infectious diseases;
7. Patients with a serious or non-healing wound, ulcer, or bone fracture;
8. Patients with CNS metastases and/or spinal cord compression, cancerous meningitis, or soft meningeal disease;
9. Patients with known to be allergic to compounds of 5-FU, capecitabine, oxaliplatin, leucovorin, or bevacizumab;
10. Patients have difficulty taking oral medication and vomit frequently.

### Randomization and blinding

Eligible patients will be randomly assigned to the placebo group or treatment group using the block randomization method, with a ratio of 1:1. The blocks are of variable sizes (2, 4, and 6) to protect concealment SAS version 9.4 statistical software (SAS Institute Inc., Cary, NC, USA) will be used to generate random sequence and an interactive web-response system (IWRS) will be used to assign patients. Investigators will be enroll patients and use identifying information to register them in the interactive web-response system. Patients will be assigned three-digit random numbers and treatment groups. HZ granule and placebo granule have identical packaging, labeling, appearance, and administration schedules. Patients, investigators, study site staff, and the sponsor will be masked to treatment assignment until the database is locked.

### Sample size

The sample size for this trial is determined based on a prior study [34]in which the median value of PFS for metastatic colorectal patients receiving placebo plus mFOLFOX6/FOLFIRI/CAPEOX and CET or BV was 6.9 months, and an estimated median PFS of 9.6 months in the HZ granule plus mFOLFOX6/FOLFIRI/CAPEOX and CET or BV, the hazard ratio was estimated as 0.71.

A total of 288 events are required for this study based on a 1:1 randomization to have an 80% power to detect a difference assuming a true hazard ratio of 0.71 of HZ granule plus mFOLFOX6/FOLFIRI/CAPEOX and CET or BV using a two-sided log-rank test at a significance level of 0.05. The overall duration of the trial plan is 36 months, with the first 24 months being the enrollment phase, and follow-up continued for about 12 months. A total sample size of 360 patients with 180 in each group is required.

### Statistical analysis

All analyses will be performed by statisticians who are independent of the random allocation of groups, using SAS version 9.4. The efficacy analyses are based on the intention-to-treat population, which included all randomized patients. A two-sided P-value of less than 0.05 is considered statistical significance. A full analysis set (FAS) includes all randomized patients who received at least one treatment as close as possible to the principle of intentional analysis. Missing data are supplemented using the LOCF (last observation carried forward) method. Per-protocol analysis set (PPS) is restricted to participants who strictly followed the provided protocol, used the trial medicine in the range of 80%-120%, and completed the study. Safety analysis set (SS) defines as all subjects who accepted at least one dose of trial medication and underwent at least one post-treatment safety assessment. Continuous variables will be expressed as mean with SD or median with quartile range and analyses are performed using the student’s test on normally distributed and homogeneity variables and Mann-Whitney’s test on non-normal variables. Enumeration data are presented as percentages and analyses by chi‐square test or Fisher exact test.

The primary endpoint of the study is PFS which will be estimated by the Kaplan-Meier method, including the median PFS and the proportion of subjects remaining progression-free at 9, 12, and 18 months. Hazard ratios and 95% confidence intervals will be presented for each group, using a log-rank test for univariate analysis and Cox proportional hazards regression for multivariable analysis.

Safety analysis will be provided the incidence of adverse events and adverse reactions that occurred in this clinical trial from baseline-normal to abnormal at the end of the study and abnormal baseline to the end of the study. The chi-squared test or Fisher test will be used to compare the incidence differences between the groups.

### Intervention

The participants will receive 6.0 g per grid of HZ granule or placebo 3 times a day based on combination chemotherapies, mFOLFOX6/FOLFIRI/CAPEOX and CET or BV until the pause of disease progression, death, withdrawal, the exhibition of unacceptable toxicity, or up to 6 months. The formula HZ granule and placebo will be supplied by Sichuan Luye Pharmaceutical CO., Ltd. (Sichuan, China). The placebo consists of maltodextrin, a bitter compound, and a natural edible pigment, which are similar to HZ granule in appearance, taste, color, smell, and weight.

According to the NCCN guidelines, the protocol for chemotherapeutic regimen is recommended by physicians, considering the patient’s need in selecting the mFOLFOX6/FOLFIRI/CAPEOX regimen, and appropriate dose adjustments will be made when patients become intolerant.

#### mFOLFOX6 regimen

mFOLFOX6 consists of 2-hour intravenous (IV) infusions of oxaliplatin at 85 mg/m^2^ and leucovorin at 400 mg/m^2^, followed by a 400 mg/m^2^ bolus infusion of 5-fluorouracil (5-FU), and then a 46- to 48-hour continuous infusion of 2400 mg/m^2^ of 5-FU with repetition of every 2 weeks.

#### FOLFIRI regimen

FOLFIRI consists of 5-FU 400 mg/m^2^ (IV bolus), leucovorin 400 mg/m^2^ and irinotecan 180 mg/m^2^, followed by a continuous 46-hour infusion of 5-FU 2400 mg/m^2^. FOLFIRI treatment cycles will be repeated every 2 weeks.

#### CAPEOX regimen

Oxaliplatin will be given at a dose of 130 mg/m^2^ through continuous IV infusion for 2 hours on the first day. Capecitabine will be taken orally twice daily in morning and evening at a dose of 1000mg/m^2^/d on days 1–14. CAPEOX chemotherapy will be administered every 3 weeks.

Based on RAS and BRAF gene detection, BV (5 mg/kg, intravenous infusion, day1, repeated in 2 weeks) or CET (500 mg/m^2^, intravenous infusion, day1, repeated in 2 weeks) will be administered in patients with wild type RAS gene and BRAF gene.

### Data management

In this study, we will record patients’ data on paper case report forms (CRFs). The clinical investigator or clinical coordinator nominated by the investigator will input the data from the study medical record to CRFs promptly and accurately. The study supervisor will make site visits to review protocol compliance, compare CRFs against individual patients’ medical records, and verify whether the drugs that are supplied, received, stored, distributed, and recovered, are recorded accordingly in accordance with relevant regulations. CRFs will be kept in a locked file cabinet that is pertinent to this research. An electronic data capture system will be used as a data management system provided by the Hospital of Chengdu University of Traditional Chinese Medicine. Data management will be performed by the investigator and monitored by an independent supervisor.

## Discussion

In recent years, chemotherapic drugs and biological agents have been used as the most effective therapies in the clinical treatment of mCRC with better outcomes of prolonged OS and PFS. However, oxaliplatin, capecitabine, BV, and CET cause a lot of adverse reactions and side effects, such as gastrointestinal reactions (nausea, vomiting, anorexia, and diarrhea) [10, 35–37], bone marrow suppression (neutropenia, peripheral sensory neuropathy, and neurotoxicity [38]) and hence, their efficacy needs to be further improved. It was reported that 20%-40% of mCRC patients receiving chemotherapy had to reduce their dose or even stop treatment due to intolerable drug-related toxicities [39, 40]. Furthermore, these negative side effects seriously affect patients’ quality of life.

CHM plays an important role in the treatment of metastatic colorectal cancer and is commonly used in clinical practice in China. Previous studies showed that CHM reduced the toxicity of chemotherapy, improved the effects of chemotherapy, promoted QOL, and relieved clinical symptoms [10]. However, there are only a few studies that concentrated on CHM combined with chemotherapy and other targeted treatment on survival outcomes in mCRC. In addition, small patient samples, non-randomized studies, and low-quality research are limitations to the development of evidence-based guidelines for using CHM in clinical practice. Hence, this prospective, multi-center, randomized, double-blinded, placebo-controlled clinical trial will investigate the benefit of HZ Granule in patients with mCRC.

HZ Granule as a classic formula of Chinese medicine is an available option for prolonging PFS, improving the quality of life of patients, and reducing or preventing chemotherapy-related AEs in patients with mCRC.

We believe that the findings of this study can be used to provide information on effective options in clinical practice.

### Ethics and dissemination

This study was approved by the Ethics Committee of the Hospital of Chengdu University of Chinese Medicine Hospital (2020KL-030). The trial was registered in the Chinese Clinical Trial Registry (ChiCTR). The final results of this study will be disseminated to the public in open-access journals and academic conferences.

The trial registration number was ChiCTR2100041643.

### Trial status

Recruitment began in July 2021, therefore 39 patients have been recruited. It is expected to finish recruiting in June 2024, 36 months in total.

## Conclusion

This study is the first step of a large-scale randomized controlled clinical trial using Chinese herbal medicine for mCRC in the province of Sichuan, China since evidence-based treatment using CHM in patients with mCRC is still lacking. The results may also provide information on effective options for the treatment of mCRC in clinical practice.

## Supporting information

S1 Checklist SPIRIT checklist. (DOC)

S1 Protocol Study protocol. (DOCX)
