# Supplementary material for: Effect of Chinese herbal medicine formula on progression-free survival among patients with metastatic colorectal cancer: Study protocol for a multi-center, double-blinded, randomized, placebo-controlled trial
Source: PLoS One. 2022 Dec 16;17(12):e0275058. doi: 10.1371/journal.pone.0275058 (PMC9757552; doi:10.1371/journal.pone.0275058)
Supplement: S1 Protocol — (DOCX) [file pone.0275058.s002.docx]

PROPOSAL

Study protocol

1. Research Title

Effect of Chinese herbal medicine formula on progression-free survival among patients with metastatic colorectal cancer: study protocol for a multi-center, double-blinded, randomized, placebo-controlled trial

2. Principal Investigator

Dr. Fengming You

3.Funding

National Administration of Traditional Chinese Medicine: 2019 Project of building evidence based practice capacity for TCM (No. 2019XZZX-ZL006).

1. Implementation Unit

Hospital of Chengdu University of Traditional Chinese Medicine

Protocol version 5 date:Sep 3, 2020

**Program Summary**

| Research Title | Effect of Chinese herbal medicine formula on progression-free survival among patients with metastatic colorectal cancer: study protocol for a multi-center, double-blinded, randomized, placebo-controlled trial |
| --- | --- |
| Roles and responsibilities | Principal Investigator: Dr. Fengming You;  Co-principal investigators: Dr. Zhuohong Li; Dr Xiaohong Shu; Dr Ying Zhang. Biostatistician: He Yao, PhD  Fengming You conceived of the study. Qiaoling Wang and Zhuohong Li initiated the study design and Xi Fu, Jing Guo and Wenyuan Li helped with implementation. |
| Study design | Prospective, multi-center, randomized, double-blinded, placebo-controlled clinical trial evaluating progression-free survival among patients with metastatic colorectal cancer. |
| Primary Objective | To evaluate the efficacy of Hezhong Granule combination with standard chemotherapy and cetuximab (CET) or bevacizumab (BV) for treating mCRC. |
| Secondary Objectives | To explore overall survival, objective response rate, safety, quality of life (QOL, and chemotherapy-induced nausea and vomiting (CINV) comparing various indicators between two groups. |
| Sample size | 360 |
| Case screening | Inclusion criteria:   1. Patients who had histologically or cytologically confirmed adenocarcinoma of the colon or rectum that was metastatic or locally advanced with stage IV; 2. Patients who underwent imaging examination (PET-CT, CT, MRI, bone scan, X-ray) that confirmed the presence of measurable lesions defined by RECIST 1.1; 3. Patients with the age ranged from 18 to 75 years; 4. Patients with Karnofsky Performance Score (KPS) of ≥50; 5. Patients who had no major surgery or open biopsy within 4 weeks of random assignment; 6. Patients who agree to receive first-line chemotherapy combined with targeted therapy (Patients may have received previous chemotherapy and/or chemoradiation per institutional standard of care. The last adjuvant therapy must have been concluded more than 6 months ago); 7. Patients with adequate organ and bone marrow functions as defined below: absolute neutrophil count (ANC) of >1.5×10^9^/L; platelets count of >100×10^9^/L; hemoglobin level of ≥90 g/L; total bilirubin level of ≤1.5 times the upper limit of normal (ULN); AST (SGOT) or ALT (SGPT) level of ≤2.5 times the ULN (or ≤5 times the ULN if it was attributable to liver metastases); Urine protein/creatinine ratio (UPCR) of <1.0. 8. Patients with the expected survival period of more than 12 weeks; 9. Patients are able to understand and willing to sign the written informed consent form. |
|  | Exclusion criteria:   1. Patients who had histologically confirmed mixed adenosquamous carcinoma with squamous cells as the main component; 2. Patients with other concurrent malignancies or had prior treatment for other carcinomas within the last 5 years, except cured carcinoma in situ of the cervix or non-melanoma skin cancer or superficial bladder tumors; 3. Patients who are pregnant or breastfeeding (a serum or urine pregnancy test is performed on all female patients who are of childbearing potential within 14 days prior to study entry); 4. Patients with prior history of hypertensive crisis, hypertensive encephalopathy, or uncontrolled hypertension (systolic blood pressure of >150mmHg, or diastolic blood pressure of >100mm Hg after anti-hypertensive therapy); 5. Patients who experienced vital organ failure or with other serious diseases including, but not limited to, coronary heart disease, cardiovascular diseases, or myocardial infarction within 12 months before being included in the study; severe neurological or psychiatric history; severe infection; active disseminated intravascular coagulation; serious diseases of the urinary system and digestive system; 6. Patients who had a combination of active hepatitis, pneumonia, and other serious infectious diseases; 7. Patients with a serious or non-healing wound, ulcer, or bone fracture; 8. Patients with CNS metastases and/or spinal cord compression, cancerous meningitis, or soft meningeal disease; 9. Patients with known to be allergic to compounds of 5-FU, capecitabine, oxaliplatin, leucovorin, or bevacizumab; 10. Patients have difficulty taking oral medication and vomit frequently. |
| Intervention | 1. Study group: The participants will receive 6.0 g per grid of HZ granule 3 times a day until the pause of disease progression, death, withdrawal, the exhibition of unacceptable toxicity, or up to 6 months. 2. Control group：The participants will receive 6.0 g per grid of placebo 3 times a day until the pause of disease progression, death, withdrawal, the exhibition of unacceptable toxicity, or up to 6 months. The placebo consists of maltodextrin, a bitter compound, and a natural edible pigment that is similar to HZ granule in appearance, taste, color, smell, and weight. 3. Standard treatment: According to the NCCN guidelines, the protocol for the chemotherapeutic regimen is recommended by physicians, considering the patient's need in selecting the mFOLFOX6/FOLFIRI/CAPEOX regimen and CET or BV.   (4)Treatment cycle: 2 weeks for 1 treatment cycle, no significant adverse effects after at least 2 cycles for long-term maintenance treatment; tumor assessments will be done every 2 months according to the standard of Response Evaluation Criteria in Solid Tumors version 1.1 (RECIST 1.1) for the initial 6 months, and then every 3 months until disease progression or death during the follow-up time. |
| Efficacy evaluation | The primary endpoint is progression-free survival (PFS). |
|  | Secondary Endpoints: overall survival, QLQ-30 scores, and CINV and adverse events of patients. |
| Statistical analysis | The primary endpoint of the study is PFS which will be estimated by the Kaplan-Meier method, including the median PFS and the proportion of subjects remaining progression-free at 9, 12, and 18 months. Hazard ratios and 95% confidence intervals will be presented for each group, using a log-rank test for univariate analysis and Cox proportional hazards regression for multivariable analysis.  Safety analysis will be provided the incidence of adverse events and adverse reactions that occurred in this clinical trial from baseline-normal to abnormal at the end of the study and abnormal baseline to the end of the study. The chi-squared test or Fisher test will be used to compare the incidence differences between the groups. |
| **Study Period** | Sep 9, 2020, to Sep 8, 2023. |

TABLE OF CONTENTS

1. BACKGROUND

1.1 Working Foundation

1. OBJECTIVES
   1. EVALUATION
2. METHODS
   1. Study design
   2. Participating centers
   3. Eligibility criteria
      1. Inclusion criteria
      2. Exclusion criteria
      3. Sample size estimation
      4. Interventions
      5. Randomization and blinding
3. PROJECT ENDPOINTS
4. DATA COLLECTION AND MANAGEMENT
5. STATISTICAL ANALYSIS
6. REGULATORY AND ETHICAL STANDARDS
   1. Ethical considerations
   2. Confidentiality
   3. Data availability statement
7. FUNDING
8. PUBLICATION
9. REFERENCES

# 1. BACKGROUND

# Colorectal cancer (CRC) is one of the most commonly diagnosed cancer worldwide and the second most deadly cancer leading to more than 935 000 deaths annually [1-2]. The incidence rate of colorectal cancer has been increasing year by year and now it ranks third among malignant tumors. It is the second most common cancer cause of death in China[3]. According to the data from Global Oncology Epidemiology Statistics 2020 (GLOBOCAN 2020), colorectal cancer is expected to account for 12.2% of all new malignancies in China in 2020[4]. Approximately 20%-30% of colorectal cancer patients present with advanced stages at the time of diagnosis, and patients in the early stages of the disease also have a 25%-50% chance of developing metastases [5-6]. In recent years, the use of cytotoxic chemotherapies and targeted agents therapies led to a significant increase in overall survival, but mCRC remains incurable in most cases [4]. Drug resistance and chemotherapy-related toxic side effects are the main causes of the failure or discontinuation of chemotherapy in mCRC. The efficiency of the first-line chemotherapic agents for primary treatment of mCRC is only 40%-60%. The efficiency of second-line chemotherapic agents is less than 30%, and the efficiency of chemotherapy in patients who are not successful with second-line chemotherapy is even lower, usually less than 15%[7]. For this group of patients who have cancer progression after second-line chemotherapy, effective treatment is lacking [8]. Furthermore, with the increase in chemotherapy cycles, the toxic side effects of chemotherapy also increase, and therefore, most patients discontinue chemotherapy because of intolerance to the toxic side effects [9]. Consequently, prolonging survival, reducing the rate of recurrence and metastasis, and improving the quality of life of patients with mCRC are problematic, and hence, alternative treatments are sought.

Chinese herbal medicine (CHM) has become a complementary alternative therapy in the treatment of cancers including CRC and its use is widely accepted in China[10-11]. CHM was shown to inhibit colon tumor formation, proliferation, migration, induce apoptosis and modulate angiogenesis of CRC cells [12-14]. In addition, many clinical trials showed that CHM used in combination with chemotherapies reduced toxicity induced by the chemotherapy, enhanced immune function, improved quality of life, and maintained safety [15-18]. Hezhong granule is a Chinese herbal formula composed of eight herbs including zingibers, ginseng, Scutellaria baiealensis, Chinese goldthread rhizome, Indian bread, Euodia rutaecarpa, and Pinellia ternata, which has been used in the treatment of mCRC for many years as an empirical formula[19-21]. However, many of the previous studies are deficient in study design, sample size, and evidence to determine whether Hezhong Granule can benefit patients with mCRC. Therefore, the current study is to observe the efficacy and safety of the Chinese herbal medicine formula, Hezhong (HZ) when combined with standard chemotherapy and molecular targeted therapy in patients with mCRC.

**1.1 Working Foundation**

The Department of Oncology at the Hospital of Chengdu University of Traditional Chinese Medicine has been committed to conducting basic and clinical research on the prevention and treatment of tumors in Chinese medicine and has conducted long-term and stable medical teaching and research collaborations with the National Cancer Institute, the University of Paris, and the Nobel Prize-winning Bishop Cancer Institute. There are 17 staff members with senior titles and 16 staff members with intermediate titles, the university has undertaken 1 national evidence-based project on TCM tumors, 10 projects of the National Natural Science Foundation of China on TCM tumors, 14 projects at the provincial level, and 4 projects at the department and bureau levels, with a total research expenditure of more than 16 million RMB.

# OBJECTIVE

The primary objective is to validate the progression-free survival (PFS) of participants with mCRC who will be randomized into the groups, HZ granule plus mFOLFOX6/FOLFIRI/CAPEOX and cetuximab (CET) or bevacizumab (BV)  versus placebo plus mFOLFOX6/FOLFIRI/CAPEOX and CET or BV. PFS is defined as the time from randomization to disease progression or death from any cause, whichever occurred earlier.

Secondary objectives are to analyze overall survival (OS, time from the start of therapy to death from any cause), objective response rate (ORR, proportion of patients with confirmed complete or partial response), safety, quality of life (QOL) [22], and chemotherapy-induced nausea and vomiting (CINV) [23], comparing various indicators between two groups.

**2.1 EVALUATION**

Tumor assessments will be done every 2 months according to the standard of Response Evaluation Criteria in Solid Tumors version 1.1 (RECIST 1.1) [24] for the initial 6 months, and then every 3 months until disease progression or death during the follow-up time. The EORTC QLQ-C30 scale will be used to assess the quality of life including functions, symptoms, and living conditions through linear conversion to a percentage system at baseline and every chemotherapy cycle, and thereafter until the end of the follow-up period [25]. Adverse events (AEs) will be recorded during the study based on the incidence, severity, and outcomes. Safety assessments is based on Common Terminology Criteria Adverse Events Version 4.0 (CTCAE v4.0) [26].

1. **METHODS**
   1. **Study design**

This study is a multi-center, randomized, double-blinded, placebo-controlled clinical trial in China. A total of 360 mCRC patients will be randomly divided into two groups (1:1). The control group will be received a placebo combined with mFOLFOX6/FOLFIRI/CAPEOX and CET or BV, a standard first-line treatment for metastatic colorectal cancer. The treatment group will receive HZ granule in combination with mFOLFOX6/FOLFIRI/CAPEOX and CET or BV.

- 1. **Study Setting**

The study plans to conduct in twelve medical institutions in China. The hospital at the Chengdu University of Traditional Chinese Medicine lead the research, and other participating units are West China Hospital of Sichuan University, Sichuan Provincial Hospital of Integrated Traditional Chinese and Western Medicine, Chengdu Hospital of Integrated Traditional Chinese and Western Medicine, Affiliated Hospital of North Sichuan Medical College, Guangyuan Central Hospital, Jintang County Hospital of Traditional Chinese Medicine, Yanjiang District Hospital of Ziyang City, Cangxi County People’s Hospital, Cangxi County Hospital of Traditional Chinese Medicine, Renshou County People’s Hospital, and Pengzhou Hospital of Traditional Chinese Medicine.

**3.3 Eligibility criteria**

Participants meet the criteria during the screening examination to be eligible to participate in the study.

**3.3.1 Inclusion Criteria**

1) Patients who had histologically or cytologically confirmed adenocarcinoma of the colon or rectum that was metastatic or locally advanced with stage IV;

2) Patients who underwent imaging examination (PET-CT, CT, MRI, bone scan, X-ray) that confirmed the presence of measurable lesions defined by RECIST 1.1;

3) Patients with the age ranged of 18 to 75 years;

4) Patients with Karnofsky Performance Score (KPS) of ≥50[27];

5) Patients who had no major surgery or open biopsy within 4 weeks of random assignment;

6) Patients who agree to receive first-line chemotherapy combined with targeted therapy (Patients may have received previous chemotherapy and/or chemoradiation per institutional standard of care. The last adjuvant therapy must have been concluded more than 6 months ago);

7) Patients with adequate organ and bone marrow functions as defined below: absolute neutrophil count (ANC) of >1.5×10^9^/L; platelets count of >100×10^9^/L; hemoglobin level of ≥90 g/L; total bilirubin level of ≤1.5 times the upper limit of normal (ULN); AST (SGOT) or ALT (SGPT) level of ≤2.5 times the ULN (or ≤5 times the ULN if it was attributable to liver metastases); Urine protein/creatinine ratio (UPCR) of <1.0.

8) Patients with the expected survival period of more than 12 weeks;

9) Patients are able to understand and willing to sign the written informed consent form.

**3.3.2 Exclusion Criteria:**

1) Patients who had histologically confirmed mixed adenosquamous carcinoma with squamous cells as the main component;

2) Patients with other concurrent malignancies or had prior treatment for other carcinomas within the last 5 years, except cured carcinoma in situ of the cervix or non-melanoma skin cancer or superficial bladder tumors;

3) Patients who are pregnant or breastfeeding (a serum or urine pregnancy test is performed on all female patients who are of childbearing potential within 14 days prior to study entry);

4) Patients with prior history of hypertensive crisis, hypertensive encephalopathy, or uncontrolled hypertension (systolic blood pressure of >150mmHg, or diastolic blood pressure of >100mm Hg after anti-hypertensive therapy);

5) Patients who experienced vital organ failure or with other serious diseases including, but not limited to, coronary heart disease, cardiovascular diseases, or myocardial infarction within 12 months before being included in the study; severe neurological or psychiatric history; severe infection; active disseminated intravascular coagulation; serious diseases of the urinary system and digestive system;

6) Patients who had a combination of active hepatitis, pneumonia, and other serious infectious diseases;

7) Patients with a serious or non-healing wound, ulcer, or bone fracture;

8) Patients with CNS metastases and/or spinal cord compression, cancerous meningitis, or soft meningeal disease;

9) Patients who are known to be allergic to compounds of 5-FU, capecitabine, oxaliplatin, leucovorin, or bevacizumab;

10) Patients have difficulty taking oral medication and vomit frequently.

**3.3.3 Sample Size**

The sample size for this trial is determined based on a prior study in which the median value of PFS for metastatic colorectal patients receiving placebo plus mFOLFOX6/FOLFIRI/CAPEOX and CET or BV was 6.9 months and the estimated risk reduction was 29% (hazard ratio of 0.71) to a median PFS of 9.6 months in the HZ granule plus mFOLFOX6/FOLFIRI/CAPEOX and CET or BV, which was clinically significant. A total of 288 events are required for this study based on a 1:1 randomization to have an 80% test power to detect a difference assuming a true hazard ratio of 0.71 of HZ granule plusmFOLFOX6/FOLFIRI/CAPEOX and CET or BV using a two-sided log-rank test at a significance level of 0.05. The overall duration of the trial plan is 36 months, with the first 24 months being the enrollment phase, and follow-up continued for about 12 months. A total sample size of 360 patients with 180 in each group is required.

**3.3.****4 Intervention**

The participants will receive 6.0 g per grid of HZ granule or placebo three times a day based on standard chemotherapy mFOLFOX6/FOLFIRI/CAPEOX and CET or BV until the pause of disease progression, death, withdrawal, the exhibition of unacceptable toxicity, or up to 6 months. The formula of HZ granule and placebo will be supplied by Sichuan Luye Pharmaceutical CO., Ltd. (Sichuan, China). The placebo consists of maltodextrin, a bitter compound, and a natural edible pigment that is similar to HZ granule in appearance, taste, color, smell, and weight.

According to the NCCN guidelines, the protocol for chemotherapeutic regimen is recommended by physicians, considering the patient's need in selecting the mFOLFOX6/FOLFIRI/CAPEOX regimen and appropriate dose adjustments will be made when patients become intolerant.

mFOLFOX6 regimen:

The FOLFOX regimen consists of 2-hour infusions of oxaliplatin at 85 mg/m^2^ and leucovorin at 400 mg/m^2^, followed by a 400 mg/m^2^ bolus infusion of fluorouracil, and then a 46- to 48-hour continuous infusion of 2400 mg/m^2^ of fluorouracil with repetition of every 2 weeks.

FOLFIRI regimen：

FOLFIRI consists of 5-FU 400 mg/m^2^ (IV bolus), leucovorin 400 mg/m^2^ and irinotecan 180 mg/m^2^, followed by a continuous 46-hour infusion of 5-FU 2400 mg/m^2^. FOLFIRI treatment cycles will be repeated every 2 weeks.

CAPEOX regimen:

Oxaliplatin will be given at a dose of 130 mg/m2 through continuous IV infusion for 2 hours on the first day. Capecitabine will be taken orally twice daily in morning and evening at a dose of 1000mg/m^2^/d on days 1-14. CAPEOX chemotherapy will be

administered every 3 weeks.

Based on RAS and BRAF gene detection, BV (5 mg/kg, intravenous infusion, 1 day, repeated in 2 weeks) or CET (500 mg/m^2^, intravenous infusion, 1 day, repeated in 2 weeks) will be administered in patients with wild type RAS gene and BRAF gene.

**3.3.5 Randomization and blinding**

Eligible patients will be randomly assigned to the placebo group or treatment group using the block randomization method, with a ratio of 1:1. The blocks are of variable sizes (2, 4, and 6) to protect concealment SAS version 9.4 statistical software (SAS Institute Inc., Cary, NC, USA) will be used to generate random sequence and an interactive web-response system (IWRS) will be used to assign patients. Investigators will be enroll patients and use identifying information to register them in the interactive web-response system. Patients will be assigned three-digit random numbers and treatment groups. HZ granule and placebo granule have identical packaging, labeling, appearance, and administration schedules. Patients, investigators, study site staff, and the sponsor will be masked to treatment assignment until the database is locked.

1. **PROJECT ENDPOINTS**

Our primary endpoint is PFS. PFS is defined as the time from randomization to disease progression or death from any cause, whichever occurred earlier. Secondary endpoints are to analyze overall survival (OS, time from the start of therapy to death from any cause), objective response rate (ORR, proportion of patients with confirmed complete or partial response), safety, quality of life (QOL), and chemotherapy-induced nausea and vomiting (CINV), comparing various indicators between two groups. Adverse events (AEs) will be recorded during the study based on the incidence, severity, and outcomes. Safety assessments are based on Common Terminology Criteria Adverse Events Version 4.0 (CTCAE v4.0).

1. **DATA COLLECTION AND MANAGEMENT**

In this study, we will record patients’ data on paper case report forms (CRFs). The clinical investigator or clinical coordinator nominated by the investigator will input the data from the study medical record to CRFs promptly and accurately. The study supervisor will make site visits to review protocol compliance, compare CRFs against individual patients’ medical records, and verify whether the drugs that are supplied, received, stored, distributed, and recovered, are recorded accordingly in accordance with relevant regulations. CRFs will keep in a locked file cabinet that is pertinent to this research. An electronic data capture system will be used as a data management system provided by the Hospital of Chengdu University of Traditional Chinese Medicine.

Data management will be performed by the investigator and monitored by an independent supervisor. Data will be reviewed for logic, data inconsistencies, and completeness.

1. **STATISTICAL ANALYSIS**

All analyses will be performed by statisticians who are independent of the random allocation of groups, using SAS version 9.4. The efficacy analyses are based on the intention-to-treat population, which included all randomized patients. A two-sided P-value of less than 0.05 is considered statistical significance. A full analysis set (FAS) includes all randomized patients who received at least one treatment as close as possible to the principle of intentional analysis. Missing data are supplemented using the LOCF (last observation carried forward) method. Per-protocol analysis set (PPS) is restricted to participants who strictly followed the provided protocol, used the trial medicine in the range of 80%-120%, and completed the study. Safety analysis set (SS) defines as all subjects who accepted at least one dose of trial medication and underwent at least one post-treatment safety [assessment](#/javascript:;). Continuous variables will be expressed as mean with SD or median with quartile range and analyses are performed using student’s test on normally distributed and homogeneity variables and Mann-Whitney’s test on non-normal variables. Enumeration data are presented as percentages and analyses by chi‐square test or Fisher exact test.

The primary endpoint of the study is PFS which will be estimated by the Kaplan-Meier method, including the median PFS and the proportion of subjects remaining progression-free at 9, 12, and 18 months. Hazard ratios and 95% confidence intervals will be presented for each group, using a log-rank test for univariate analysis and Cox proportional hazards regression for multivariable analysis. Binary outcomes will be analyzed by a two-sample comparison of proportions using chi-square test. Continuous variables will be tested either by the student’s t test or by the Wilcoxon rank sum test depending whether assumptions for the t-test are satisfied.

Safety analysis will be provided the incidence of adverse events and adverse reactions that occurred in this clinical trial from baseline-normal to abnormal at the end of the study and abnormal baseline to the end of the study. The chi-squared test or Fisher test will be used to compare the incidence differences between the groups.

All subgroup analyses will be considered exploratory. Comparison of the outcomes by sex (male versus female), age group (age≤65 versus >65 years), and smoking status(former versus current versus never) will be performed. This analysis will be planned and described in statistical analysis plan (SAP).

Primary and secondary outcomes will be adjusted for covariates. All adjusted analysis will be exploratory. Co-variates of interest will be included based on clinical relevance and will be specified in SAP. Adjustment will be performed by adding covariates to the original models.

1. **ETHICAL STANDARDS**
   1. **Ethical considerations**

This study will be conducted in conformance with ICH guideline for Good Clinic Practice and the principles of the Declaration of Helsinki or the laws and regulations of the country.

This protocol, the ICFs, and relevant supporting information must be submitted to the IRB and must be approved by the IRB. This study was reviewed by the Ethics Committee of the Hospital of Chengdu University of Chinese Medicine Hospital. The trial will be registered in the Chinese Clinical Trial Registry (ChiCTR). The final results of this study will be disseminated to the public in open-access journals and academic conferences.

- 1. **Confidentiality**

The project personnel will maintain all records, patient files, and other source data for the time periods required by regulatory authorities. All patient information will be stored on a high-security computer system and kept strictly confidential. Patient medical information obtained as a result of this study is considered confidential and disclosure to third parties is prohibited.

**7.3 Data availability statement**

The data that support the findings of this study are available from the corresponding

author upon reasonable request.

Figure 1 Flow chat.


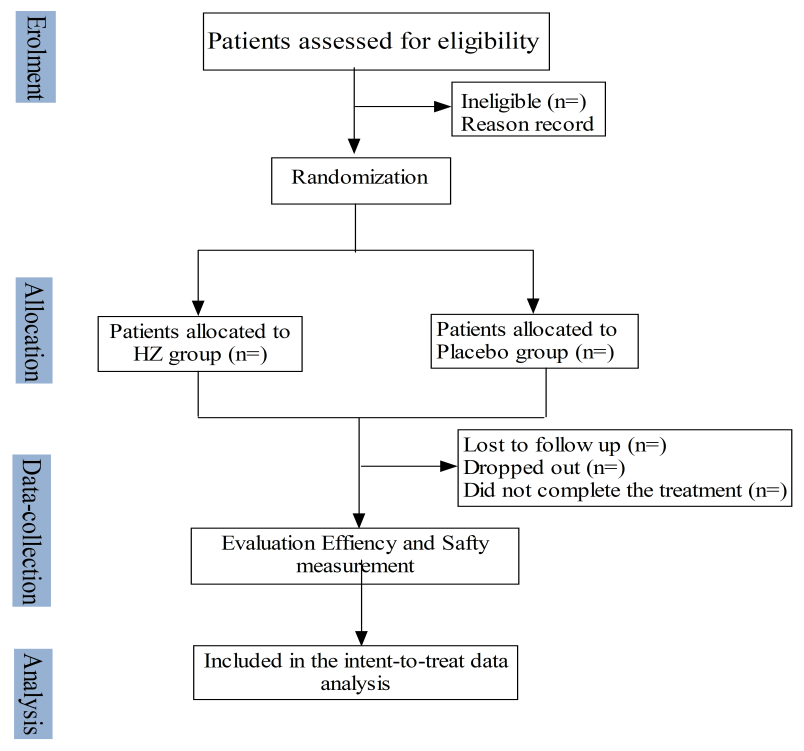


1. **Funding**

National Administration of Traditional Chinese Medicine: 2019 Project of building evidence based practice capacity for TCM (No. 2019XZZX-ZL006) will provide the funding for the trial drug and placebo used for this trial. The cost of the 360 patients’ recruitment also will be provided by this funding. The HeZhong granule and placebo will be manufactured by Sichuan Luye Pharmaceutical CO., Ltd. (Sichuan, China).

1. **PUBLICATION**

The results of this study will be published or presented at open-access journals and academic conferences in a timely, objective, and clinically meaningful manner.

1. **REFERENCES**

GBD 2017 Colorectal Cancer Collaborators. The global, regional, and national burden of colorectal cancer and its attributable risk factors in 195 countries and territories, 1990-2017: a systematic analysis for the Global Burden of Disease Study 2017. Lancet Gastroenterol Hepatol. 2019 Dec;4(12):913-933.

Dekker E, Tanis PJ, Vleugels JL A, Kasi PM, Wallace MB. Colorectal cancer. Lancet 2019;394:1467-1480.

Feng RM, Zong YN, Cao SM, Xu RH. Current cancer situation in China: good or bad news from the 2018 Global Cancer Statistics? Cancer Commun. 2019 Apr;39(1):22.

Sung H, Ferlay J, Siegel R, Laversanne M, Soerjomataram I, Jemal A, Bray F. Global cancer statistics 2020: GLOBOCAN estimates of incidence and mortality worldwide for 36 cancers in 185 countries. CA Cancer J Clin. 2021 May;71(3):209-249.

Mody K, Baldeo C, Bekaii-Saab T. Antiangiogenic Therapy in Colorectal Cancer. Cancer journal. 2018 Jul/Aug;24(4): 165-170.

6. Benoist C, Christophe T, Franck B. Therapeutic strategy in unresectable metastatic colorectal cancer:An updated review. Ther Adv Med Oncol. 2015 May;7(3):153-169.

Modesta DP, Pant S , Sartore-Bianchi A. Treatment sequencing in metastatic colorectal cancer. European Journal of Cancer. 2019 Mar;109:70-83.

Geng F, Wang Z, Yin H, Yu J, CaoB. Molecular Targeted Drugs and Treatment of Colorectal Cancer: Recent Progress and Future Perspectives. Cancer Biother Radiopharm. 2017 Jun;32(5):149-160.

Goldberg R, Sargent D, Morton R, Fuchs C, Ramanathan R, Williamson S, Findlay B, Pitot H, Alberts S. Randomized controlled trial of reduced-dose bolus fluorouracil plus leucovorin and irinotecan or infused fluorouracil plus leucovorin and oxaliplatin in patients with previously untreated metastatic colorectal cancer: a North American Intergroup Trial. J Clin Oncol. 2006 Jul;24(21):3347-3353.

Lam W, Bussom S, Guan F, Jiang Z, Zhang W, Gullen EA, Liu SH, Cheng YC. The four-herb Chinese medicine PHY906 reduces chemotherapy-induced gastrointestinal toxicity. Sci Transl Med. 2010 Aug;2(45):45-59.

[Zhang](https://pubmed.ncbi.nlm.nih.gov/?sort=date&term=Zhang+X&cauthor_id=34421064) XY, Qiu H, [L](https://pubmed.ncbi.nlm.nih.gov/?sort=date&term=Li+C&cauthor_id=34421064)i CS, [Cai](https://pubmed.ncbi.nlm.nih.gov/?sort=date&term=Cai+P&cauthor_id=34421064) PP, [Qi](https://pubmed.ncbi.nlm.nih.gov/?sort=date&term=Qi+F&cauthor_id=34421064) FH. The positive role of traditional Chinese medicine as an adjunctive therapy for cancer. Biosci Trends. 2021,15(5):283-298.

12. Liang YC, Tsai SH, Chen L, Lin-Shiau SY, Lin JK. Resveratrol-induced g2 arrest through the inhibition of cdk7 and p34cdc2 kinases in colon carcinoma ht29 cells. Bio-chem Pharmacol. 2003 Apr;65(7): 1053-1060.

13. Juan ME, Wenzel U, Daniel H, Planas JM. Resveratrol induces apoptosis through ros-dependent mitochondria pathway in ht-29 human colorectal carcinoma cells. J Agric Food Chem. 2008 Jun; 56(12): 4813-4818.

Mok T S K, Yeo W, Johnson P J, Hui P, Ho W M, Lam K C , Xu M, Chak K, Chan A ,Wong H, Mo F, Zee B. A double-blind placebo-controlled randomized study of Chinese herbal medicine as complementary therapy for reduction of chemotherapy-induced toxicity. Annals of Oncology. 2007 Apr;18(4):768-774.

Fuggetta MP, Lanzilli G, Tricarico M, [Cottarelli](https://pubmed.ncbi.nlm.nih.gov/?term=Cottarelli+A&cauthor_id=16918129) A, [Falchetti](https://pubmed.ncbi.nlm.nih.gov/?term=Falchetti+R&cauthor_id=16918129) R, [Ravagnan](https://pubmed.ncbi.nlm.nih.gov/?term=Ravagnan+G&cauthor_id=16918129) G, [Bonmassar](https://pubmed.ncbi.nlm.nih.gov/?term=Bonmassar+E&cauthor_id=16918129) E. Effect of resveratrol on proliferation and telomerase activity of human colon cancer cells in vitro. J Exp Clin Cancer Res. 2006 Jun; 25(2): 189-193.

[Zhang](https://pubmed.ncbi.nlm.nih.gov/?term=Zhang+T&cauthor_id=28840585) T, [Yang](https://pubmed.ncbi.nlm.nih.gov/?term=Yang+YF&cauthor_id=28840585) Y F, [He](https://pubmed.ncbi.nlm.nih.gov/?term=He+B&cauthor_id=28840585) B, [Yi](https://pubmed.ncbi.nlm.nih.gov/?term=Yi+DH&cauthor_id=28840585) DH, [Hao](https://pubmed.ncbi.nlm.nih.gov/?term=Hao+J&cauthor_id=28840585) J, [Zhang](https://pubmed.ncbi.nlm.nih.gov/?term=Zhang+D&cauthor_id=28840585)D. Efficacy and Safety of Quxie Capsule in Metastatic Colorectal Cancer: A Double-Blind Randomized Placebo Controlled Trial. Chin J Integr Med. 2018 Mar;24(3):171-7.

[Li](https://pubmed.ncbi.nlm.nih.gov/?sort=date&term=Li+L&cauthor_id=34950221) LX, [Wang](https://pubmed.ncbi.nlm.nih.gov/?sort=date&term=Wang+Y&cauthor_id=34950221) YJ, [Huang](https://pubmed.ncbi.nlm.nih.gov/?sort=date&term=Huang+X&cauthor_id=34950221) XW, [Sun](https://pubmed.ncbi.nlm.nih.gov/?sort=date&term=Sun+J&cauthor_id=34950221) J, [Zhang](https://pubmed.ncbi.nlm.nih.gov/?sort=date&term=Zhang+J&cauthor_id=34950221) J. Effect of Xiaoyutang Combined with Intraperitoneal Heat Perfusion Chemotherapy on Immune Function, Circulating mir, Prognosis, and Survival of Postoperative Patients with Colorectal Cancer. Comput Math Methods Med. 2021 Dec;2021:1619809.

Jia R, Liu NN, Cai GX, Zhang Y, Xiao HJ, Zhou LH , [Ji](https://pubmed.ncbi.nlm.nih.gov/?term=Ji+Q&cauthor_id=33747930) Q, [Zhao](https://pubmed.ncbi.nlm.nih.gov/?term=Zhao+L&cauthor_id=33747930) L, [Zeng](https://pubmed.ncbi.nlm.nih.gov/?term=Zeng+P&cauthor_id=33747930) P H, [Liu](https://pubmed.ncbi.nlm.nih.gov/?term=Liu+H&cauthor_id=33747930) HM, [Huo](https://pubmed.ncbi.nlm.nih.gov/?term=Huo+J&cauthor_id=33747930) JG, [Yue](https://pubmed.ncbi.nlm.nih.gov/?term=Yue+X&cauthor_id=33747930) XQ, [Zhang](https://pubmed.ncbi.nlm.nih.gov/?term=Zhang+Y&cauthor_id=33747930) Y, [Wu](https://pubmed.ncbi.nlm.nih.gov/?term=Wu+C&cauthor_id=33747930) CJ, [Sun](https://pubmed.ncbi.nlm.nih.gov/?term=Sun+X&cauthor_id=33747930) XT, [Feng](https://pubmed.ncbi.nlm.nih.gov/?term=Feng+Y&cauthor_id=33747930) YY, [Liu](https://pubmed.ncbi.nlm.nih.gov/?term=Liu+H&cauthor_id=33747930) H J, [Liu](https://pubmed.ncbi.nlm.nih.gov/?term=Liu+H&cauthor_id=33747930) H, [Han](https://pubmed.ncbi.nlm.nih.gov/?term=Han+Z&cauthor_id=33747930) ZF, [Lai](https://pubmed.ncbi.nlm.nih.gov/?term=Lai+Y&cauthor_id=33747930) YY, [Zhang](https://pubmed.ncbi.nlm.nih.gov/?term=Zhang+Y&cauthor_id=33747930) YB, [Han](https://pubmed.ncbi.nlm.nih.gov/?term=Han+G&cauthor_id=33747930) G, [Gong](https://pubmed.ncbi.nlm.nih.gov/?term=Gong+H&cauthor_id=33747930) HJ, [Wang](https://pubmed.ncbi.nlm.nih.gov/?term=Wang+Y&cauthor_id=33747930) Y, [Li](https://pubmed.ncbi.nlm.nih.gov/?term=Li+Q&cauthor_id=33747930) Q. Effect of PRM1201 Combined With Adjuvant Chemotherapy on Preventing Recurrence and Metastasis of Stage III Colon Cancer:A Randomized, Double-Blind, Placebo-Controlled Clinical Trial. Front Oncol. 2021 Mar;11:618793.

Curra M, Soares Junior LAV, Martins MD, Santos PSDS. Chemotherapy protocols and incidence of oral mucositis. An integrative review. Einstein (Sao Paulo). 2018 Apr;16(1):eRW4007.

Li YJ, Qiu SG, Liang J. Clinical Study on Banxia Xiexin Decoction in Treatment of Disharmony between Liver and Spleen after Colorectal Cancer Surgery. World Chinese Medicine. 2017 Jul;12(7)1523-1530.

Zhao LM, Liu MS, Shu ZF. Clinical Study on Modified Banxia Xiexin Tang Combined with FOLFOX Scheme for Colorectal Cancer. Journal of New Chinese medicine. 2021 Jul;53(14)117-120.

Niezgoda HE, Pater JL. A validation study of the domains of the core EORTC quality of life questionnaire. Qual Life Res. 1993 Oct;2(5):319-25.

Grunberg SM, Osoba D, Hesketh PJ, [Gralla](https://pubmed.ncbi.nlm.nih.gov/?term=Gralla+RJ&cauthor_id=15599601) R J, [Borjeson](https://pubmed.ncbi.nlm.nih.gov/?term=Borjeson+S&cauthor_id=15599601) S, [Rapoport](https://pubmed.ncbi.nlm.nih.gov/?term=Rapoport+BL&cauthor_id=15599601) B L, [Bois](https://pubmed.ncbi.nlm.nih.gov/?term=du+Bois+A&cauthor_id=15599601) A, [Tonato](https://pubmed.ncbi.nlm.nih.gov/?term=Tonato+M&cauthor_id=15599601) M. Evaluation of new antiemetic agents and definition of antineoplastic agent emetogenicity-An update. Support Care Cancer. 2005 Feb;2: 80-84.

Eisenhauer EA, Therasse P, Bogaerts J, [Schwartz](https://pubmed.ncbi.nlm.nih.gov/?term=Schwartz+LH&cauthor_id=19097774) LH, [Sargent](https://pubmed.ncbi.nlm.nih.gov/?term=Sargent+D&cauthor_id=19097774) D, [Ford](https://pubmed.ncbi.nlm.nih.gov/?term=Ford+R&cauthor_id=19097774) R, [Dancey](https://pubmed.ncbi.nlm.nih.gov/?term=Dancey+J&cauthor_id=19097774) J, [Arbuck](https://pubmed.ncbi.nlm.nih.gov/?term=Arbuck+S&cauthor_id=19097774) S, [Gwyther](https://pubmed.ncbi.nlm.nih.gov/?term=Gwyther+S&cauthor_id=19097774) S, [Mooney](https://pubmed.ncbi.nlm.nih.gov/?term=Mooney+M&cauthor_id=19097774) M, [Rubinstein](https://pubmed.ncbi.nlm.nih.gov/?term=Rubinstein+L&cauthor_id=19097774) L, [Shankar](https://pubmed.ncbi.nlm.nih.gov/?term=Shankar+L&cauthor_id=19097774) L, [Dodd](https://pubmed.ncbi.nlm.nih.gov/?term=Dodd+L&cauthor_id=19097774) L, [Kaplan](https://pubmed.ncbi.nlm.nih.gov/?term=Kaplan+R&cauthor_id=19097774) R, [Lacombe](https://pubmed.ncbi.nlm.nih.gov/?term=Lacombe+D&cauthor_id=19097774) D, [Verweij](https://pubmed.ncbi.nlm.nih.gov/?term=Verweij+J&cauthor_id=19097774) J. New response evaluation criteria in solid tumours: revised RECIST guideline (version 1.1). Eur J Cancer. 2009 Jan;45(2)228-247.

Wong CK, Guo VY, Chen J, Lam CL. Methodological and reporting quality of comparative studies evaluating health-related quality of life of colorectal cancer patients and controls: a systematic review. Dis Colon Rectum. 2016 Nov; 59(11): 1073-1086.

National Cancer Institute. Common Terminology Criteria for Adverse Events.

Version 4.0. NCI 2010. Accessed on 28 June 2019.

Hwang SS, Scott CB, Chang VT, Cogswell J, Srinivas S, Kasimis B. Prediction of survival for advanced cancer patients by recursive partitioning analysis: role of Karnofsky performance status, quality of life, and symptom distress. Cancer Invest. 2004;22(5):678–687.

Hurwitz H, Fehrenbacher L, Novotny W, Cartwright T, Hainsworth J, Heim W, [Berlin](https://pubmed.ncbi.nlm.nih.gov/?term=Berlin+J&cauthor_id=15175435) J, [Baron](https://pubmed.ncbi.nlm.nih.gov/?term=Baron+A&cauthor_id=15175435) A, [Griffing](https://pubmed.ncbi.nlm.nih.gov/?term=Griffing+S&cauthor_id=15175435) S, [Holmgren](https://pubmed.ncbi.nlm.nih.gov/?term=Holmgren+E&cauthor_id=15175435) E, [Ferrara](https://pubmed.ncbi.nlm.nih.gov/?term=Ferrara+N&cauthor_id=15175435) N, [Fyfe](https://pubmed.ncbi.nlm.nih.gov/?term=Fyfe+G&cauthor_id=15175435) G, [Rogers](https://pubmed.ncbi.nlm.nih.gov/?term=Rogers+B&cauthor_id=15175435) B, [Ross](https://pubmed.ncbi.nlm.nih.gov/?term=Ross+R&cauthor_id=15175435) R,  [Kabbinavar](https://pubmed.ncbi.nlm.nih.gov/?term=Kabbinavar+F&cauthor_id=15175435) F. Bevacizumab plus Irinotecan, Fluorouracil, and Leucovorin for Metastatic Colorectal Cancer. N Engl J Med. 2004 Jun;350(23):2335-2342.

Kuebler JP, Wieand HS, O’Connell MJ, Smith RE, Colangelo LH, Yothers G, [Petrelli](https://pubmed.ncbi.nlm.nih.gov/?term=Petrelli+NJ&cauthor_id=17470851) NJ, [Findlay](https://pubmed.ncbi.nlm.nih.gov/?term=Findlay+MP&cauthor_id=17470851) MP, [Seay](https://pubmed.ncbi.nlm.nih.gov/?term=Seay+TE&cauthor_id=17470851) TE, [Atkins](https://pubmed.ncbi.nlm.nih.gov/?term=Atkins+JN&cauthor_id=17470851) JN, [Zapas](https://pubmed.ncbi.nlm.nih.gov/?term=Zapas+JL&cauthor_id=17470851) JL, [Goodwin](https://pubmed.ncbi.nlm.nih.gov/?term=Goodwin+JW&cauthor_id=17470851) JW, [Fehrenbacher](https://pubmed.ncbi.nlm.nih.gov/?term=Fehrenbacher+L&cauthor_id=17470851) L, [Ramanathan](https://pubmed.ncbi.nlm.nih.gov/?term=Ramanathan+RK&cauthor_id=17470851) RK, [Conley](https://pubmed.ncbi.nlm.nih.gov/?term=Conley+BA&cauthor_id=17470851) BA, [Flynn](https://pubmed.ncbi.nlm.nih.gov/?term=Flynn+PJ&cauthor_id=17470851) PJ, [Soori](https://pubmed.ncbi.nlm.nih.gov/?term=Soori+G&cauthor_id=17470851) G, [Colman](https://pubmed.ncbi.nlm.nih.gov/?term=Colman+LK&cauthor_id=17470851) LK, [Levine](https://pubmed.ncbi.nlm.nih.gov/?term=Levine+EA&cauthor_id=17470851) EA, [Lanier](https://pubmed.ncbi.nlm.nih.gov/?term=Lanier+KS&cauthor_id=17470851) KS, [Wolmark](https://pubmed.ncbi.nlm.nih.gov/?term=Wolmark+N&cauthor_id=17470851) N. Oxaliplatin combined with weekly bolus fluorouracil and leucovorin as surgical adjuvant chemotherapy for stage II and III colon cancer: results from NSABP C-07. J Clin Oncol. 2007 Jun;25(16):2198-2204.

Kidwell KM, Yothers G, Ganz PA, Land SR, Ko CY, Cecchini RS, [Kopec](https://pubmed.ncbi.nlm.nih.gov/?term=Kopec+JA&cauthor_id=22569841) JA,  [Wolmark](https://pubmed.ncbi.nlm.nih.gov/?term=Wolmark+N&cauthor_id=22569841) N.Long-term neurotoxicity effects of oxaliplatin added to fluorouracil and leucovorin as adjuvant therapy for colon cancer: results from National Surgical Adjuvant Breast and Bowel Project trials C-07 and LTS-01. Cancer. 2012 Nov;118

:5614-5622.

André T, Boni C, Navarro M, Tabernero J, Hickish T, Topham C, [Bonetti](https://pubmed.ncbi.nlm.nih.gov/?term=Bonetti+A&cauthor_id=19451431) A, [Clingan](https://pubmed.ncbi.nlm.nih.gov/?term=Clingan+P&cauthor_id=19451431) P, [Bridgewater](https://pubmed.ncbi.nlm.nih.gov/?term=Bridgewater+J&cauthor_id=19451431) J, [Rivera](https://pubmed.ncbi.nlm.nih.gov/?term=Rivera+F&cauthor_id=19451431) F, [Gramont](https://pubmed.ncbi.nlm.nih.gov/?term=de+Gramont+A&cauthor_id=19451431) A.Improved overall survival with oxaliplatin, fluorouracil, and leucovorin as adjuvant treatment in stage II or III colon cancer in the MOSAIC trial. J Clin Oncol. 2009 Jun;27(19):3109-3116.

Gao J, He Q, Li Y, Shen L, Hua D, Mao J. Clinical and T. Oncology:Polymorphism of TS 3’-UTR predicts survival of Chinese advanced gastric;cancer patients receiving first-line capecitabine plus paclitaxel. Clin Transl Oncol. 2013 Aug;15(8):619-625.

Kotaka M, Yamanaka T, Yoshino T, Manaka D, Eto T, Hasegawa J, Takagane A, Nakamura M, Kato T, Munemoto Y, Nakamura F, Bando H, Taniguchi H, Gamoh M, Shiozawa M, Saji S, Maehara Y, Mizushima T, Ohtsu A, Mori M. Safety data from the phase III Japanese ACHIEVE trial: part of an international, prospective, planned pooled analysis of six phase III trials comparing 3 versus 6 months of oxaliplatin-based adjuvant chemotherapy for stage III colon cancer. ESMO open. 2018 Apr;3(3):e000354.
